# NuA3 HAT antagonizes the Rpd3S and Rpd3L HDACs to optimize mRNA and lncRNA expression dynamics

**DOI:** 10.1093/nar/gkaa781

**Published:** 2020-10-03

**Authors:** Ji Hyun Kim, Chae Young Yoon, Yukyung Jun, Bo Bae Lee, Ji Eun Lee, So Dam Ha, Hyeonju Woo, Ahyoung Choi, Sanghyuk Lee, Woojin Jeong, Ji Hyung Kim, TaeSoo Kim

**Affiliations:** Department of Life Science and the Research Center for Cellular Homeostasis, Ewha Womans University, Seoul 03760, Korea; Department of Life Science and the Research Center for Cellular Homeostasis, Ewha Womans University, Seoul 03760, Korea; Ewha-JAX Cancer Immunotherapy Research Center, Ewha Womans University, Seoul 03760, Korea; Department of Life Science and the Research Center for Cellular Homeostasis, Ewha Womans University, Seoul 03760, Korea; Department of Life Science and the Research Center for Cellular Homeostasis, Ewha Womans University, Seoul 03760, Korea; Department of Life Science and the Research Center for Cellular Homeostasis, Ewha Womans University, Seoul 03760, Korea; Department of Life Science and the Research Center for Cellular Homeostasis, Ewha Womans University, Seoul 03760, Korea; Department of Bio-Information Science, Ewha Womans University, Seoul, 03760, Korea; Ewha-JAX Cancer Immunotherapy Research Center, Ewha Womans University, Seoul 03760, Korea; Department of Bio-Information Science, Ewha Womans University, Seoul, 03760, Korea; Department of Life Science and the Research Center for Cellular Homeostasis, Ewha Womans University, Seoul 03760, Korea; Department of Biotechnology, College of Life Sciences and Biotechnology, Korea University, Seoul 02841, Korea; Department of Life Science and the Research Center for Cellular Homeostasis, Ewha Womans University, Seoul 03760, Korea

## Abstract

In yeast, NuA3 histone acetyltransferase (NuA3 HAT) promotes acetylation of histone H3 lysine 14 (H3K14) and transcription of a subset of genes through interaction between the Yng1 plant homeodomain (PHD) finger and H3K4me3. Although NuA3 HAT has multiple chromatin binding modules with distinct specificities, their interdependence and combinatorial actions in chromatin binding and transcription remain unknown. Modified peptide pulldown assays reveal that the Yng1 N-terminal region is important for the integrity of NuA3 HAT by mediating the interaction between core subunits and two methyl-binding proteins, Yng1 and Pdp3. We further uncover that NuA3 HAT contributes to the regulation of mRNA and lncRNA expression dynamics by antagonizing the histone deacetylases (HDACs) Rpd3S and Rpd3L. The Yng1 N-terminal region, the Nto1 PHD finger and Pdp3 are important for optimal induction of mRNA and lncRNA transcription repressed by the Set2-Rpd3S HDAC pathway, whereas the Yng1 PHD finger–H3K4me3 interaction affects transcriptional repression memory regulated by Rpd3L HDAC. These findings suggest that NuA3 HAT uses distinct chromatin readers to compete with two Rpd3-containing HDACs to optimize mRNA and lncRNA expression dynamics.

## INTRODUCTION

Covalent modifications of histones, including acetylation, methylation, phosphorylation and ubiquitination play essential roles in eukaryotic transcription. Histone acetylation promotes RNA Polymerase II (RNA Pol II) transcription by inducing the open chromatin structure and disrupting the interaction between DNA and histones. In addition, this modification generates binding sites for factors that regulate chromatin structure and transcription. Histone acetylation is a highly dynamic modification that is regulated by the antagonistic function of histone acetyltransferases (HATs) and histone deacetylases (HDACs) (1,2). Histone methylation at specific sites also affects histone acetylation by targeting HATs and HDACs or by enhancing their activity (3).

Co-transcriptional histone H3 methylations at K4 and K36 regulate histone acetylation at distinct regions of genes (3,4). Whereas H3K4me3 by Set1-COMPASS is enriched at active promoters, H3K4me2 and H3K4me1 peak at 5′ and 3′ transcribed regions, respectively (2). Several HATs or HDACs bind to H3K4me3 or H3K4me2 (5). Three yeast ING (inhibitor of growth) proteins, Yng1 (NuA3 HAT), Yng2 (NuA4 HAT) and Pho23 [Rpd3 large (Rpd3L) HDAC] strongly bind to H3K4me3 to maintain optimal acetylation patterns at promoters (5). In 5′ transcribed regions, Set3 HDAC binds to H3K4me2 (6,7). Set2-mediated H3K36me3 peaks at 3′ ends of genes and recruits and/or activates the Rpd3 small (Rpd3S) HDAC (8,9). The Eaf3 and Rco1 subunits of Rpd3S HDAC are required for the interaction between Rpd3S HDAC and H3K36-methylated nucleosome and histone deacetylation (10). Recent studies revealed that multiple HDACs have little effect on global transcript levels under steady-state growth conditions, but mainly affect the kinetics of gene induction or repression upon environmental changes. The Set3 HDAC and Set2-Rpd3S HDAC pathway cooperate with lncRNA transcription to delay induction of mRNA transcription (6,11). Instead, histone deacetylation at active promoters mediated by interaction between H3K4me3 and Rpd3L HDAC contributes to transcriptional repression memory (TREM) for faster gene repression (12).

NuA3 HAT includes six subunits: Sas3, Yng1, Nto1, Eaf6, Taf14 and Pdp3 (13–17). The catalytic subunit Sas3 primarily acetylates K14 of histone H3 (14,18,19). This complex has multiple chromatin binding modules that bind to unmodified histones, H3K4me3 and H3K36me3, and chromatin binding of NuA3 HAT requires both H3K4 and H3K36 methylation (5,16,17,20–22). The Yng1 PHD finger strongly binds to H3K4me3 (5,16,21), whereas its N-terminal region mediates interaction with unmodified histone tails (20). A PHD finger of Nto1 is known to weakly bind to H3K36me3 *in vitro* (5). Taf14, a member of several complexes including NuA3 HAT, SWI/SNF, Ino80, RSC and RNA Pol II (14,23–26), recognizes H3K9 acetylation and histone crotonylation via its YEATS domain (27,28). A recent study identified Pdp3 as a subunit of NuA3 HAT and showed that its PWWP domain directly binds to H3K36me3 (17). Although the interaction between specific histone modifications and individual subunits of NuA3 HAT is well understood, their combinatorial action in chromatin binding remains unclear. The exact function of NuA3 HAT in transcription is also largely unknown. The Yng1 PHD finger binding to H3K4me3 enhances H3K14 acetylation by NuA3 HAT, but loss of this interaction causes defects in transcription of only a subset of genes (16). Furthermore, mutants for NuA3 HAT do not have a strong effect on global gene expression patterns (29).

Here, we show that the Yng1 N-terminal region is critical for the integrity of NuA3 HAT, as loss of this region caused dissociation of two methyl-binding proteins, Yng1 and Pdp3, from the complex. In addition, we also find that NuA3 HAT is important for regulation of mRNA and lncRNA expression dynamics. Although basal transcript levels were not affected, mutation in the Yng1 N-terminal region, Nto1 PHD finger and Pdp3 of NuA3 HAT reduced or delayed mRNA and lncRNA induction repressed by the Set2-Rpd3S HDAC during carbon source shifts. By contrast, interaction between the Yng1 PHD finger and H3K4me3 was specific for competing with Rpd3L HDAC to fine-tune TREM. We therefore propose that NuA3 HAT plays opposing roles of two Rpd3-containing HDACs to optimize mRNA and lncRNA expression dynamics using its distinct chromatin readers.

## MATERIALS AND METHODS

### Yeast strains and culture conditions

Yeast strains used in this study are listed in Supplementary Table S1. The time course experiments were carried out with indicated strains as previously described (6,11). To generate Yng1 N-terminal deletion mutant, *yng1* (W180A) and *nto1* (Q264T, A265Y) mutants, the *delitto perfetto* strategy was used (30). The sequences of oligonucleotides used in this study are listed in Supplementary Table S2.

### Peptide pull down analysis

Whole cell extracts were prepared with binding buffer (50 mM Tris-HCl (pH 7.5), 0.1% NP-40) containing 150–300 mM NaCl and protease inhibitors. Two hundred and fifty micrograms of whole cell extracts was incubated with 1 μg of biotinylated histone peptides (Anaspec) and 25 μl of streptavidin coupled Dynabeads (Invitrogen) at 4°C for 4 h. Beads were washed five times with 1.5 ml of binding buffer and precipitates were resolved by SDS-PAGE followed by immunoblot analysis.

### Chromatin immunoprecipitations (ChIPs)

Chromatin immunoprecipitations (ChIPs) were done as previously described (7) with oligonucleotides listed in Supplementary Table S2. The following antibodies were used: 1 μl anti-H3 (Abcam Ab1791) and 2 μl anti-acetyl H3K14 (Millipore 07–353). Binding for anti-H3 or for anti-acetyl H3K14 was done overnight in FA lysis buffer containing 275 mM NaCl. Precipitates were washed with the same buffer, once with FA lysis buffer containing 500 mM NaCl, once with 10 mM Tris-HCl(pH 8.0), 0.25 M LiCl, 1 mM EDTA, 0.5% NP-40, 0.5% Na deoxycholate, and once with TE (10 mM Tris-HCl[pH 8.0], 1 mM EDTA). Precipitated DNAs were analyzed by real-time quantitative PCR (qPCR) using CFX96 cycler (Bio-Rad) and THUNDERBIRD SYBR qPCR Mix (TOYOBO).

### Reverse transcription and qPCR analysis

RNA was extracted from cells with hot phenol. Total RNAs was treated with DNase I (Thermo Fisher Scientific) and first-strand cDNA was prepared with 1 μg of total RNA, ReverTra Ace® qPCR RT kit (TOYOBO), and gene-specific primers. cDNA was analyzed by real-time qPCR using THUNDERBIRD SYBR qPCR Mix (TOYOBO) and CFX96 cycler (Bio-Rad). The sequences of oligonucleotides used in this study are listed in Supplementary Table S2.

### Western blot analysis

Cells expressing TAP-tagged or myc-tagged proteins were grown in YPD to mid-log phase. Cells were lysed using lysis buffer (50 mM Tris, pH 7.5, 150 mM NaCl, 0.1% NP-40) with protease inhibitors (Pepstatin A 1 μM, Aprotinine 0.3 μM, Leupeptin 1 μM, PMSF 1 mM) and glass beads. Protein concentration was quantitated by Bradford assay. For SDS-PAGE and western blot analyses, 15–30 μg of whole cell extracts was used. Proteins were separated in SDS-PAGE and transferred onto a PVDF membrane (Millipore). The blots were visualized on film with SuperSignal West Pico Chemiluminescent Substrate (Thermo Fisher Scientific). Anti-TAP (Sigma, P1291) or anti-Myc (BioLegend, 626802) was used for western blot analysis.

### Northern blot analysis

Total RNA was isolated from cells with hot phenol. Ten micrograms of total RNA was separated on an agarose gel and then transferred to nylon membrane (Bio-Rad). Northern blot analysis was done as previously described (31). The sequences of oligonucleotides used for northern blot analysis are listed in Supplementary Table S2. Strand-specific probes were produced by unidirectional PCR in the presence of [α-^32^P] dATP with only one primer. Hybridization was done in a buffer containing 1% BSA, 7% SDS, 1 mM EDTA (pH 8.0) and 300 mM sodium phosphate buffer (pH 7.2). The membranes were washed with 2× SSC and 0.1% SDS for 20 min and exposed to PhosphoImager.

### Co-immunoprecipitations

Whole cell extracts were prepared with binding buffer (50 mM Tris-HCl (pH 7.5), 0.1% NP-40) containing 150 mM NaCl and protease inhibitors (Pepstatin A 1 μM, Aprotinine 0.3 μM, Leupeptin 1 μM, PMSF 1 mM). Three hundred and fifty micrograms of total extracts were incubated with 25 μl of IgG Sepharose (GE Healthcare) at 4°C for 4 h. After binding, beads were washed five times with 1.5 ml of binding buffer and precipitates were resolved by SDS-PAGE followed by immunoblot analysis.

### Spot assay

For spotting analysis, cells were resuspended at 2.5 × 10^8^/ml and subjected to 3-fold serial dilutions in synthetic complete (SC) media lacking any carbon sources. Three microliters of each dilution was spotted on the indicated plates.

### RNA sequencing and data analysis

Sequencing libraries were constructed using the TruSeq Stranded Total RNA Library Prep Kit (Illumina) after ribosomal RNA was depleted using the Ribo-Zero yeast kit (Epicenter) according to the manufacturer’s instructions. Transcriptome sequencing was performed with Illumina NextSeq 500 sequencing platform for 101-mer paired-end reads. Raw sequencing data were filtered using Sickle v1.33 (https://github.com/najoshi/sickle) with reads below a quality score of Q20 and a length <50 nucleotides removed. The reads were aligned to the *Saccharomyces cerevisiae* reference genome (EF4, obtained from Ensembl release 74) using TopHat v2.0.13 (32). For strand-specific alignment, we used the option of –fr-firststrand and supplied the sample-specific values of insert size (i.e. average and standard deviation) that were obtained from the Bowtie alignment on the same scaffold with concordant directions. The transcript abundance was analyzed by Cufflinks v2.2.1 (33) and Fragment Per Kilobase of transcript per Million mapped reads (FPKM) was used as a normalized expression level.

### ChIP-Seq data analysis

The MNase-based ChIP-Seq data sets for Sas3–6HA and histone methylation were downloaded from the GEO database, accession numbers: GSE93059 (22) and GSE61888 (34), respectively. All sequencing reads were aligned to the *S. cerevisiae* genome using Burrow-Wheeler Aligner (BWA) v0.7.5a (35) with the default parameters and filtered using Samtools with reads below a mapping quality score of 10. After mapping, the read counts were converted to coverage wig files using the ngs.BaseAlignCounts function of java-genomic-toolkit (https://github.com/timpalpant/java-genomics-toolkit). Average enrichment profiles were generated using the Cis-regulatory Element Annotation System (CEAS) v1.0.2 (36).

## RESULTS

### Combinatorial or distinct actions of the chromatin binding modules of NuA3 HAT

**Figure 1.**
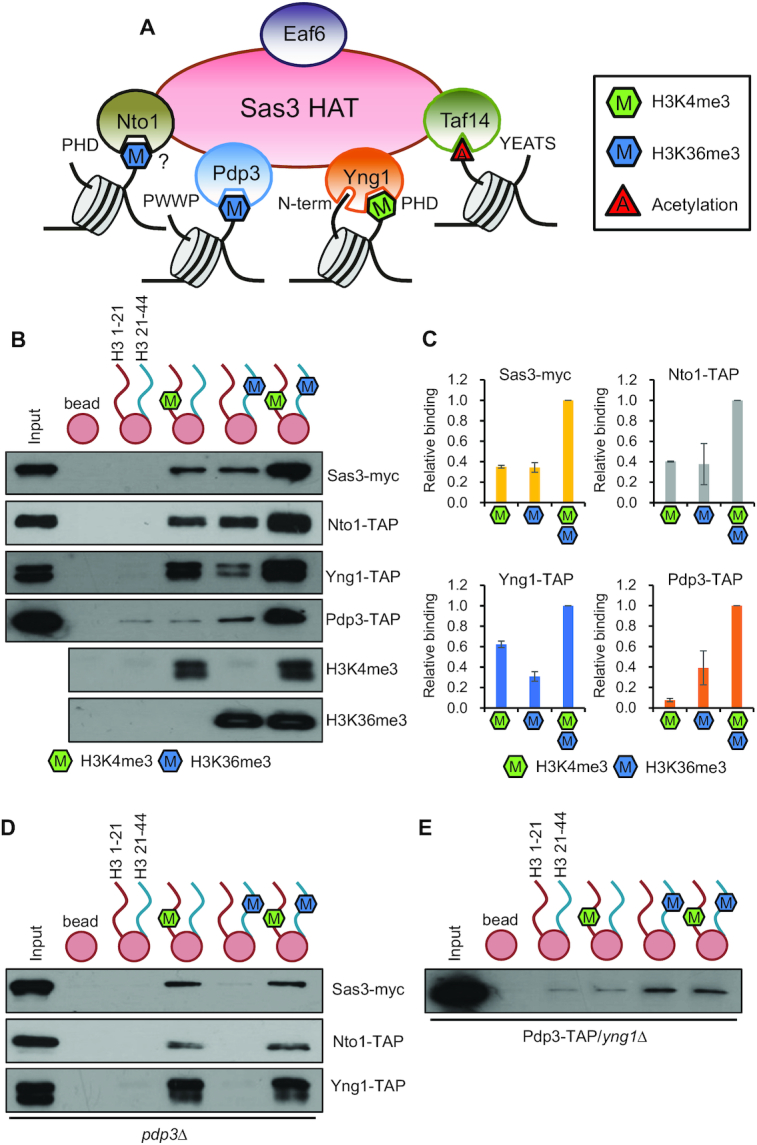
NuA3 HAT exists in several distinct complexes. (**A**) Schematic representation of multiple chromatin binding domains in NuA3 HAT. Whereas Yng1 PHD finger binds to H3K4me3, its N-terminal region interacts with unmodified histone tails. NuA3 HAT also binds to H3K36me3 via the Pdp3 PWWP domain and the Nto1 PHD finger domain. In addition, the Taf14 YEATS domain is known to bind to histone acetylation. (**B**) Interdependence and distinct roles of chromatin binding modules of NuA3 HAT. Histone peptide pulldown assays were performed with whole cell extracts from the indicated strains and 1 μg of histone peptides immobilized on magnetic beads in binding buffer containing 250 mM NaCl. Equal amount (0.5 μg of each) of two histone peptides, H3 1–21 and H3 21–44, methylated on K4 or K36 or not was used. Precipitated proteins were analyzed by immunoblot analyses with anti-myc or anti-TAP antibodies. Histone methylation on K4 or K36 was confirmed by immunoblot analyses with anti-H3K4me3 or anti-H3K36me3 antibodies. Two independent experiments showed the same results. (**C**) Quantitation from (B). Error bars show the standard deviation (S.D.) calculated from two independent experiments. (**D**) Pdp3 is important for NuA3 HAT binding to H3K36me3. Peptide pulldown assay was done as in (B). Two independent experiments showed the same results. (**E**) Loss of Yng1 causes dissociation of Pdp3 from H3K4me3. Peptide pulldown assay was performed as in (B). Two independent experiments showed the same results.

Although NuA3 HAT has multiple chromatin binding subunits, and the interaction between specific histone modifications and individual subunits is well known, their interdependence or combinatorial actions remains elusive (13,16,17,21,27) (Figure 1A). To test this, we performed peptide binding assays using whole cell extracts and histone H3 peptides, H3 amino acids 1–21 methylated on K4 (H3K4me3) or H3 amino acids 21–44 trimethylated on K36 (H3K36me3). As shown in Figure 1B, equal amounts of both peptides, H3 1–21 and H3 22–44, trimethylated (or not) on K4 and K36, respectively, were conjugated with beads to precipitate NuA3 HAT. Both Sas3-myc and Nto1-TAP showed stronger binding to methylated peptides than to beads alone or unmodified histone peptides (Figure 1B). Furthermore, they showed comparable binding efficiency to both H3K4me3 and H3K36me3. This binding was significantly increased when both peptides trimethylated on either K4 or K36 were present (Figure 1B). Interestingly, Yng1-TAP and Pdp3-TAP showed distinct binding patterns. Although their binding to histone peptides was increased in the presence of the two methylated peptides, Yng1-TAP and Pdp3-TAP preferentially bound to K4 and K36 methylated peptides, respectively (Figure 1B and C). In addition, Pdp3 had a weak binding affinity to unmodified histone tails (Figure 1B).

We next tested the effect of loss of Yng1 or Pdp3 on binding of NuA3 HAT to histone peptides. Upon deletion of *PDP3*, no binding of Sas3-myc, Nto1-TAP and Yng1-TAP to H3K36me3 was seen (Figure 1D). In contrast, their binding to H3K4me3 was unaffected. Furthermore, no increase in binding was seen when the two peptides were added (Figure 1D). Similar patterns were also observed for Pdp3. Loss of Yng1 had no effect on Pdp3 binding to H3K36me3 (Figure 1E). These results suggest that chromatin binding modules of NuA3 HAT function independently and/or together to mediate chromatin association of this complex.

### The N-terminal region of Yng1 is important for the integrity of NuA3 HAT

**Figure 2.**
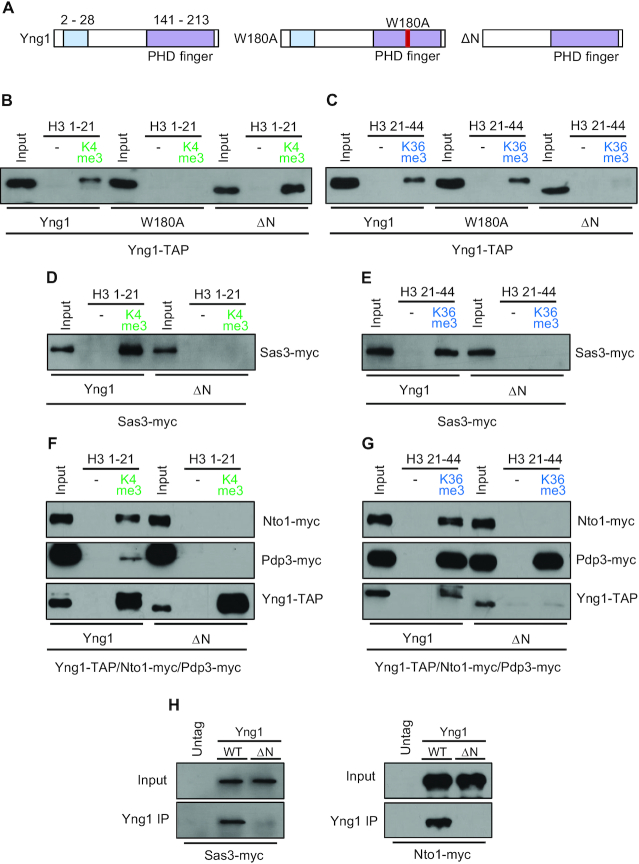
Yng1 N-terminal region bridges NuA3 HAT core and two methyl-binders. (**A**) Schematic representation of two chromatin binding domains in Yng1: the Yng1 N-terminal region (amino acids 2–28) and the Yng1 PHD finger (amino acids 141–213). The W180A mutation in PHD finger or deletion of N-terminal region (ΔN) was created by the *delitto perfetto* strategy. (**B**) Yng1 binding to H3K4me3 requires its PHD finger but not N-terminal region. Histone peptide pulldown assays were performed with binding buffer containing 300 mM NaCl as in Figure 1B. Two independent experiments showed the same results. (**C**) The Yng1 N-terminal region is important for association between Yng1 and H3K36me3. Histone peptide pulldown assay was done in binding buffer containing 250 mM NaCl. Two independent experiments showed the same results. (**D** and**E**) Interaction between Sas3 and H3K4me3 or H3K36me3 is absent in ΔN strains. Peptide pulldown assay was done as in (C). Two independent experiments showed the same results. (**F**) Nto1 fails to bind to H3K4me3 and H3K36me3 in the absence of the Yng1 N-terminal region. Peptide pulldown assay was done as in (C). Two independent experiments showed the same results. (**G**) Only Pdp3 but not Nto1 binds to H3K36me3 in ΔN strains. Peptide pulldown assay was done as in (C). Two independent experiments showed the same results. (**H**) Loss of the Yng1 N-terminal region disrupts interaction between Yng1 and Sas3 or Nto1. Co-immunoprecipitation assays were carried out whole cell extracts from the indicated strains with Yng1-TAP and IgG beads in binding buffer containing 150 mM NaCl. Precipitated proteins were analyzed by immunoblot analyses with anti-myc or anti-TAP antibodies. Two independent experiments showed the same results.

To further examine the roles of each subunit of NuA3 HAT in chromatin binding, peptide binding assays were performed using cell extracts from various mutants. Yng1 has at least two chromatin binding domains, the N-terminal region that binds to unmodified histones and a PHD finger that recognizes H3K4me3 (16,20,21) (Figure 2A). As reported, wild-type Yng1 strongly bound to the peptide methylated on K4 of histone H3, and this binding was absent when a tryptophan within the methyl-binding pocket of the PHD finger was mutated to alanine (W180A) (Figure 2B). However, Yng1 lacking N-terminal region (ΔN) showed slightly higher binding to H3K4me3 than wild-type Yng1 (Figure 2B). Since the N-terminal region binds to unmodified histones, we also performed peptide binding assays with unmethylated H3 peptides (20). Wild-type Yng1 showed strong binding to H3 peptide under low salt conditions, but this binding was significantly reduced when N-terminal region was deleted (Supplementary Figure S1A). Nto1 has two PHD finger domains with distinct specificities. The first PHD finger (PHD1) has weak affinity for H3K36me3 but the second one (PHD2) binds to unmodified histone tails *in vitro* (5) (Supplementary Figure S1B). Although mutations in PHD1 of Nto1 (Q264T/A265Y) decreased its binding to H3K36me3, we did not observe a reduction in binding of Nto1 (Supplementary Figure S1C). Instead, loss of Pdp3 abrogated Nto1 binding to H3K36me3. Nto1 binding was detected in wild type cells but not in *pdp3Δ*, indicating that Pdp3 is a major reader of H3K36me3 in NuA3 HAT (Figure 1C and Supplementary Figure S1D). Nto1 showed a weak binding to unmodified histone peptides under low salt conditions, and mutations in PHD1 decreased this interaction (Supplementary Figure S1E). Interestingly, deletion of *NTO1* reduced the levels of Sas3 and Yng1 but not of Pdp3 (Supplementary Figure S1F). Nto1 seems to affect the stability of these proteins because no change in transcription levels of *SAS3* and *YNG1* was seen in *nto1Δ* (29).

As shown in Figure 1B, Yng1 had a weak binding to H3K36me3 in wild-type cells and the interaction was not changed in the *yng1* W180A mutant. Unexpectedly, loss of the Yng1 N-terminal region caused a complete loss of this binding (Figure 2C). To further confirm this result, we carried out peptide binding assays for Sas3-myc and other subunits of NuA3 in wild-type and ΔN mutant cells. Sas3 binding to H3K4me3 and H3K36me3 was observed in wild-type cells but not in ΔN mutant (Figure 2D and E). In addition, although Yng1 binding to H3K4me3 was not affected, Nto1 and Pdp3 failed to bind to H3K4me3 in the ΔN mutant consistent with the results obtained for Sas3 (Figure 2F). Deletion of the N-terminal region of Yng1 caused failure of Nto1 and Yng1 to bind to H3K36me3, whereas it did not affect Pdp3 binding to H3K36me3 (Figure 2G). These findings suggest that the Yng1 N-terminal region is likely important for the association between the methyl-binding proteins, Yng1 and Pdp3, and the rest of NuA3 HAT. To monitor the interaction between Sas3 and Yng1 or between Nto1 and Yng1, we performed co-immunoprecipitation assays in wild-type and ΔN mutant cells. Consistent with the peptide binding patterns, Yng1 co-immunoprecipitated with Sas3 and Nto1 in wild-type cells but not in ΔN mutant (Figure 2H). Taken together, these results strongly argue that the Yng1 N-terminal region is critical for the integrity of NuA3 HAT. Specifically, loss of the Yng1 N-terminal region causes defects in the association between NuA3 HAT core components and two methyl-readers, Yng1 and Pdp3.

### Multiple chromatin readers of NuA3 HAT except the Yng1 PHD finger antagonize the Set2-Rpd3S HDAC pathway

Although NuA3 HAT is important for histone H3 K14 and K23 acetylation, the role of individual chromatin binding subunits of NuA3 HAT in transcription is not fully understood. We and other groups have shown that chromatin modifiers primarily affect the kinetics of gene induction or repression upon environmental changes (4,6,11,12,37–39). H3K36 methylation by Set2 is localized within transcribed regions and targets histone deacetylation by the Rpd3S HDAC to slow elongation and repress transcription initiation from cryptic promoters (8,9,11,40). Upon environmental shifts, approximately 60 mRNAs and 335 cryptic transcripts were strongly induced in the absence of this pathway (11). However, the HATs counteracting this pathway remain to be identified. NuA3 HAT is a potential candidate as it recognizes H3K36 methylation via Pdp3 and its chromatin binding requires H3K36 methylation by Set2 (17,22) (Figure 1B and D).

**Figure 3.**
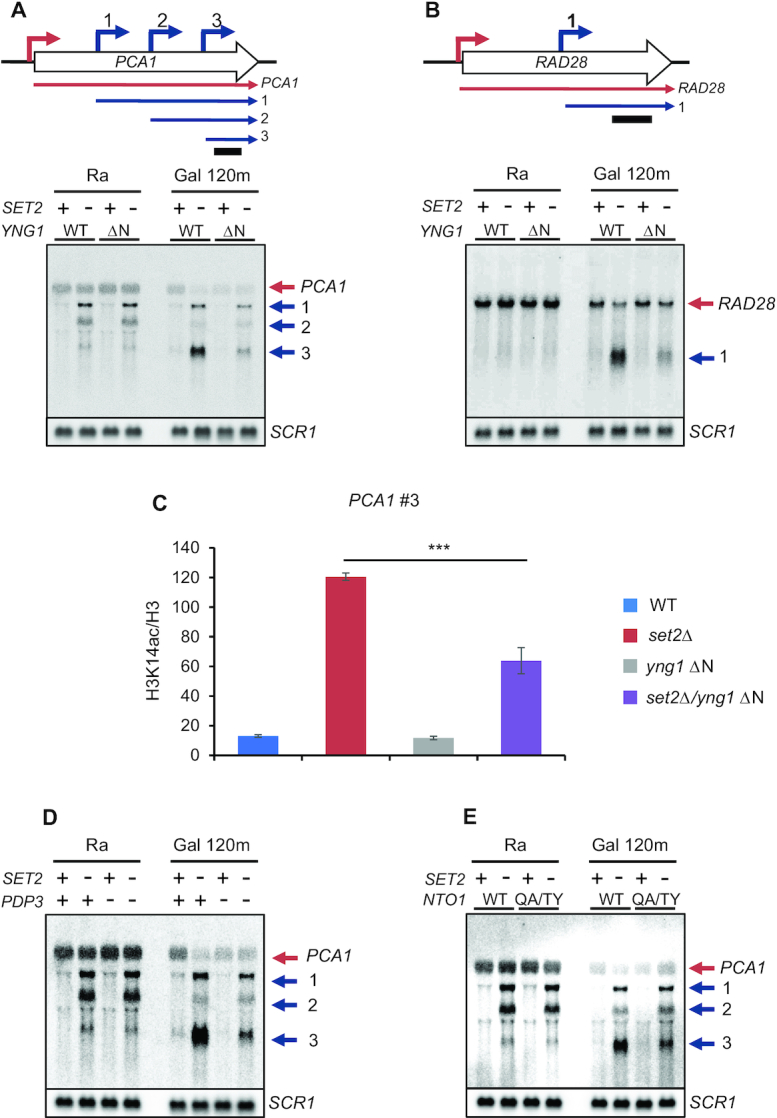
NuA3 HAT positively regulates inducible cryptic promoters repressed by Set2. (**A** and**B**) Galactose-inducible cryptic promoters of *PCA1* and *RAD28* are positively regulated by NuA3 HAT. Northern blot analysis was performed with 3′-strand specific DNA probe. The indicated cells were grown in synthetic complete (SC) medium containing raffinose (Ra) and shifted to SC-galactose media for 120 min (Gal 120m). Bottom panels show cryptic transcripts of *PCA1* (A) or *RAD28* (B) detected by northern blot analysis, which are schematicized at top. Red arrows are core promoters and blue arrows are cryptic promoters that produce short cryptic transcripts. A bar underneath upper panels indicates position of DNA probe used for northern blot analysis. *SCR1* was used as a loading control. Two independent experiments showed the same results. (**C**) NuA3 HAT acetylates histone H3 at *PCA1* cryptic promoter. Cross-linked chromatin from the indicated strains grown in YPD was precipitated with anti-H3 or anti-acetyl H3K14. PCR analysis was carried out on the galactose-inducible cryptic promoter of *PCA1*. A non-transcribed region near the telomere of chromosome VI was used for an internal control. The signals for acetyl H3K14 were quantitated and normalized to total H3 signal, and the ratios were graphed. Error bars show the standard deviation (S.D.) calculated from three biological replicates, each with three technical replicates. ***P< 0.001 (two-tailed unpaired Student's *t* tests). (**D** and**E**) Pdp3 and Nto1 are required for full activation of galactose-inducible cryptic promoters of *PCA1* and *RAD28*. Northern blot analysis with the indicated strains was done as in (A). Two independent experiments showed the same results.

To test this hypothesis, we carried out northern blot analyses to monitor inducible cryptic transcripts of *PCA1* and *RAD28* during carbon source shifts. Cells were initially grown in media containing raffinose and then transferred to galactose for 120 min. *PCA1* has three cryptic promoters that respond differentially to galactose exposure in mutants for the Set2-Rpd3S pathway (11) (Figure 3A). The first cryptic promoter (#1) close to the core promoter was activated when *SET2* is deleted, but it was not affected by carbon source shifts. By contrast, the second (#2) and third (#3) promoters were down- and upregulated during galactose incubation, respectively (Figure 3A). Loss of the Yng1 N-terminal region in *set2Δ* background had no effect on cryptic transcript levels in raffinose medium. Furthermore, transcript levels from the first and the second cryptic promoters were similar to those of *set2Δ* upon galactose exposure (Figure 3A). A similar pattern was observed for *STE11* cryptic transcripts, which are not sensitive to distinct carbon sources (Supplementary Figure S2A). However, cryptic transcripts from the third promoter induced by galactose in *set2Δ* were significantly reduced in *set2Δ*/*yng1* ΔN mutants (Figure 3A). The *RAD28* cryptic transcript induced by galactose in *set2Δ* was also downregulated significantly in *set2Δ*/*yng1* ΔN mutants (Figure 3B).

We next analyzed NuA3 HAT binding to 335 inducible cryptic promoters regulated by the Set2-Rpd3S pathway using a published data (22). Sas3–6HA showed weak enrichment in transcribed regions of all yeast genes, and a similar pattern with increased binding was observed for Set2-sensitive inducible cryptic promoters (Supplementary Figure S2B). To test whether NuA3 HAT is required for H3 acetylation at these promoters, chromatin immunoprecipitation (ChIP) assay with an antibody recognizing acetylated K14 of H3 was performed. Loss of Set2 strongly increased H3K14 acetylation at *PCA1* cryptic promoter but this acetylation was reduced in *set2Δ*/*yng1* ΔN mutants, indicating that NuA3 HAT directly binds to inducible cryptic promoters to acetylate histone H3 (Figure 3C). In addition to the Yng1 N-terminal region, Pdp3 and the Nto1 PHD finger were required for full activation of the *PCA1* cryptic promoter (#3) in *set2Δ* or *rco1Δ* background (Figure 3D,E and Supplementary Figure S2C). Unexpectedly, the *PCA1* cryptic promoter (#1) was differentially regulated by Set2 and Rco1. Although cryptic transcripts from this promoter were slightly increased in *rco1Δ* during galactose exposure, this pattern was not seen in *SET2* deleting cells (Figure 3A,D and Supplementary Figure S2C). Surprisingly, the interaction between the Yng1 PHD finger and H3K4me3 was dispensable as indicated by a slight increase of *PCA1* cryptic transcript levels in *set2Δ*/*yng1* W180A mutants (Supplementary Figure S2D). Taken together, these results suggest that multiple chromatin binding modules of NuA3 HAT, except the Yng1 PHD finger, contribute to full activation of inducible cryptic promoters repressed by the Set2-Rpd3S pathway.

### NuA3 HAT fine-tunes mRNA expression dynamics

**Figure 4.**
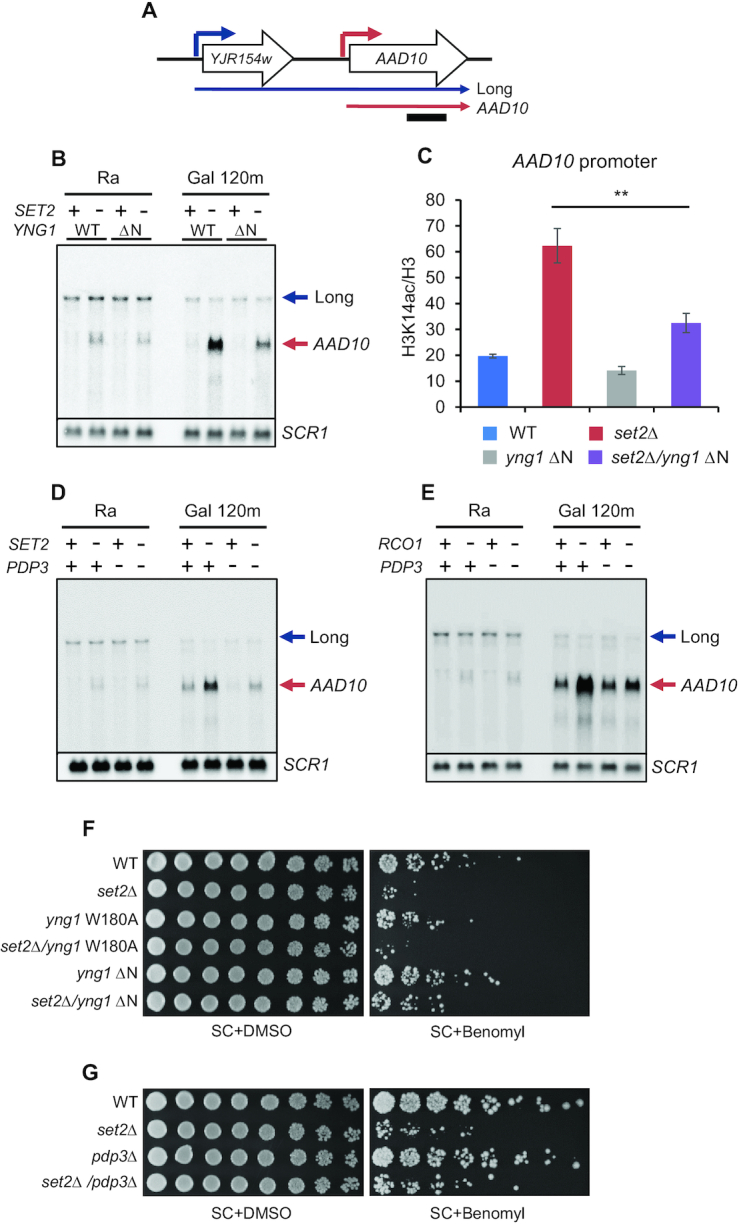
NuA3 HAT antagonizes the Set2-Rpd3S pathway. (**A**) Blue and red arrow indicate a distal promoter that produces a lncRNA and a core promoter for *AAD10* mRNA, respectively. A bar underneath indicates position of probe used for northern blot analysis. (**B**) Loss of the Yng1 N-terminal region attenuates *AAD10* induction in *SET2* deleting cells. Northern blot analysis of *AAD10* was performed with a 3′-strand specific DNA probe. The indicated cells were grown in synthetic complete (SC) medium containing raffinose (Ra) and shifted to SC-galactose media for 120 min (Gal 120m). *SCR1* was used as a loading control. Two independent experiments showed the same results. (**C**) NuA3 HAT acetylates histones at the *AAD10* promoter. ChIP assay was performed as in Figure 3C. **P< 0.01 (two-tailed unpaired Student's *t* tests). (**D** and**E**) Pdp3 is required for *AAD10* induction in mutants for the Set2-Rpd3S pathway. Northern blot analysis of *AAD10* was carried out as in (**B**). (**F**) Loss of the Yng1 N-terminal region but not its PHD finger mutation partially suppresses the growth defect of *SET2* deleting cells in the presence of Benomyl. The indicated strains were spotted in 3-fold dilutions on synthetic complete (SC) medium containing DMSO (2 days growth shown) or 50 μg/ml Benomyl (5.6 days growth shown). (**G**) Growth defect of *set2Δ* is partially suppressed by deletion of *PDP3*. Spot assay of the indicated strains was done as in (F).

In addition to cryptic promoters, H3K36 methylation by Set2 and Rpd3S HDAC can target mRNA promoters by overlapping lncRNA transcription to modulate mRNA expression dynamics (11,37,41,42). We, therefore, tested whether NuA3 HAT also contributes to the Set2-Rpd3S pathway-mediated regulation of mRNA expression. *AAD10* is overlapped with lncRNA transcribed from an upstream promoter (11) (Figure 4A). Upon loss of Set2, lncRNA transcript levels were not changed but *AAD10* mRNA was significantly increased during galactose incubation (Figure 4B). By contrast, *YNR068C*, which also has an overlapping lncRNA from the upstream gene, *BSC5*, showed no increase in transcript levels upon galactose exposure (Supplementary Figure S3A). Deletion of the Yng1 N-terminal region in *set2Δ* background caused significant downregulation of *AAD10*, but not *YNR068C* mRNA, during galactose incubation, indicating that NuA3 HAT mainly affects expression of inducible mRNA genes (Figure 4B and Supplementary Figure S3A). Consistent with this, H3K14 acetylation levels at the *AAD10* promoter were also reduced in *set2Δ*/*yng1* ΔN mutant compared with *set2Δ* (Figure 4C). Loss of Pdp3 in *set2Δ* or in *rco1Δ* background and *nto1* mutation (Q264T/A265Y in PHD finger) in *set2Δ* also caused the reduction of *AAD10* mRNA expression compared with the effect of a single deletion (Figure 4D,E and Supplementary Figure S3B). It should be noted that Pdp3 may function independently of H3K36 methylation by Set2 as Pdp3 was required for full activation of *AAD10* mRNA and *PCA1* cryptic transcription even in *SET2* deleting cells (Figures 3D and 4D). As seen for *PCA1* cryptic transcripts, the Yng1 PHD finger was not required for full activation of *AAD10* mRNA (Supplementary Figure S3C). Therefore, except for the Yng1 PHD finger, multiple chromatin readers of NuA3 HAT contribute to Set2-Rpd3S pathway-mediated regulation of mRNA expression dynamics.

Set2 is known to affect cell cycle progression by controlling expression of cell cycle-regulated genes via overlapping lncRNA transcription, and loss of Set2 confers sensitivity to a microtubule inhibitor, benomyl (37). Since NuA3 HAT antagonizes the function of Set2, we examined whether loss of NuA3 HAT suppresses this phenotype. As reported, deletion of *SET2* reduced the resistance to benomyl (Figure 4F). Mutations in *YNG1* (W180A or ΔN) had no detectable phenotypes in the presence of benomyl. However, *yng1* ΔN, but not the W180A mutation, slightly suppressed the growth defect of *set2Δ* in benomyl-containing media (Figure 4F). This suppression was also observed when *PDP3* was deleted (Figure 4G). These findings suggest that NuA3 HAT partially affects cell cycle progression by contributing to Set2-mediated control of cell cycle-regulated genes.

**Figure 5.**
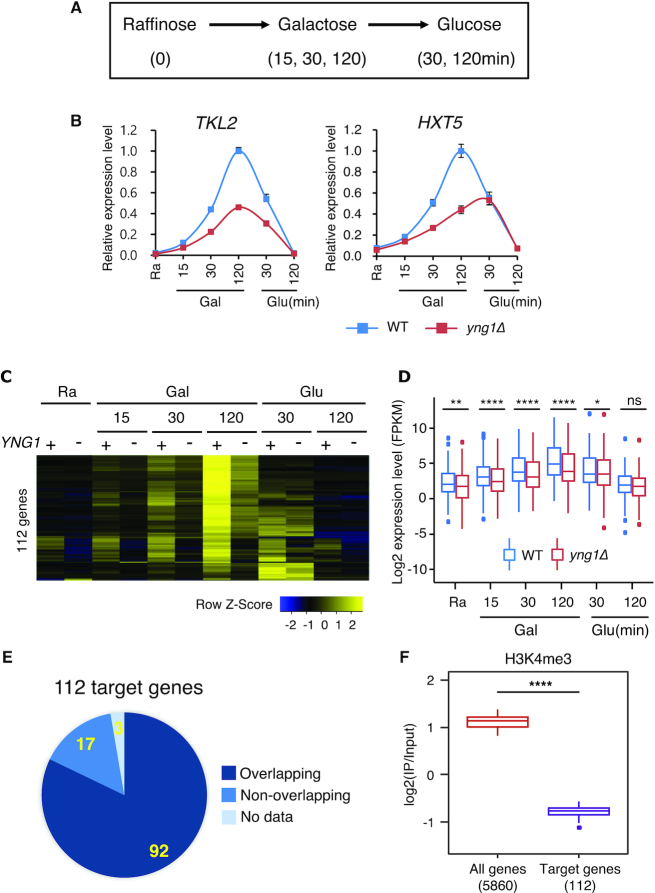
NuA3 HAT fine-tunes the kinetics of transcriptional induction. (**A**) Schematic representation of the time course experiments to monitor changes in transcript levels upon carbon source shifts. (**B**) RNA samples from the time course experiments in (A) were analyzed by RT-PCR. *SCR1* was used as an internal control. Error bars show the standard deviation (S.D.) calculated from two biological replicates, each with three technical replicates. (**C**) Two RNA samples from the time course experiments in (B) were analyzed by strand-specific RNA sequencing. NuA3 HAT-activated genes were identified as those showing at least 1.7-fold decrease in transcript levels at one more time points and the *P*-value from Cuffdiff < 0.05. (**D**) Averaged profiles of expression signals of 112 genes in (C); ns: not significant, *P < 0.05, **P < 0.01, ***P < 0.001, and ****P < 0.0001 (paired *t*-test). (**E**) A pie chart shows the number of genes (yellow) with overlapping lncRNA transcription. Among these, 82% (92 genes) of genes are overlapped with lncRNA transcription. (**F**) H3K4me3 is depleted at promoters of NuA3 HAT target genes. The average enrichment of H3K4me3 for all genes (red) and for 112 genes from (C; purple). H3K4me3 pattern was analyzed using the data sets from Weiner *et al.* (34). ****P < 0.0001 (paired *t*-test).

To further explore the role of NuA3 HAT in transcription, gene expression during carbon source shifts was analyzed in wild-type cells and mutants for NuA3 HAT (Figure 5A). *TKL2* and *HXT5* are induced during galactose incubation and repressed by glucose in wild-type cells. In addition, they are negatively regulated by the Set2-Rpd3S pathway (6,11). Loss of Yng1 caused delayed and/or reduced induction of these genes upon galactose exposure (Figure 5B). This pattern was also observed in the *yng1* ΔN, *nto1Δ* and *nto1* PHD mutants (Supplementary Figure S4A–C). However, the W180A mutation in the Yng1 PHD finger had no effect on induction (Supplementary Figure S4D). To investigate how NuA3 HAT affects gene expression dynamics, total RNAs isolated from wild-type and *yng1Δ* cells during carbon source shifts were analyzed by strand-specific RNA-sequencing (Figure 5A). NuA3-regulated genes were identified as those showing a 1.7-fold decrease in quantitated gene expression levels at one or more time points (the *P*-value from Cuffdiff < 0.05). We identified 112 genes that were positively regulated by NuA3 HAT (Figure 5C and D). Interestingly, approximately 82% of NuA3-regulated genes were overlapped with lncRNA transcription (Figure 5E). Consistent with the observation that the *yng1* W180A mutation had no effect on gene induction, H3K4me3 was depleted at the promoters of NuA3 target genes (Figure 5F). Therefore, NuA3 HAT might function with lncRNA transcription to fine-tune mRNA expression dynamics.

### Interaction between the Yng1 PHD finger and H3K4me3 contributes to TREM

Targeting of NuA3 HAT to promoters via interaction between the Yng1 PHD finger and H3K4me3 promotes histone acetylation and transcription of a subset of genes (16). Furthermore, other ING family proteins that bind to H3K4me3 are essential for the function of multiple chromatin modifying complexes (12,43–45). However, the W180A mutation in the Yng1 PHD finger had no effect on mRNA and lncRNA expression dynamics (Supplementary Figures S2D, S3C and S4D). This is likely attributed to lack of H3K4me3 at target promoters (Figure 5F).

**Figure 6.**
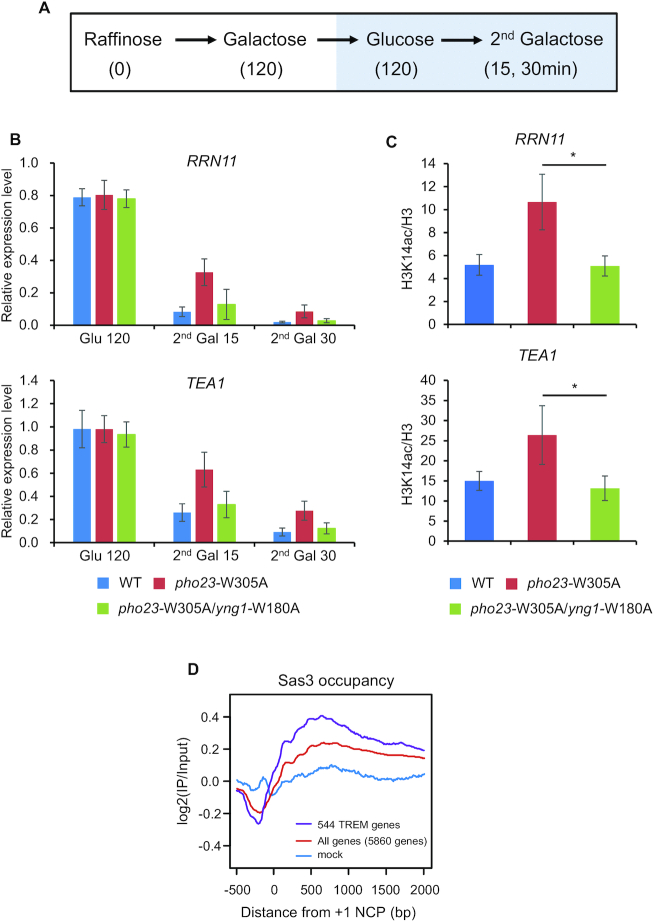
Interaction between the Yng1 PHD finger and H3K4me3 is important for TREM. (**A**) Schematic representation of the time course experiments to monitor changes in transcript levels upon carbon source shifts. (**B**) Loss of the Yng1 PHD finger–H3K4me3 interaction facilitates gene repression in mutant for *PHO23*. RNA samples at glucose 120 min (Glu 120), second galactose 15 min (second Gal 15) and second galactose 30 min (second Gal 30) from (A) were analyzed by RT-PCR. *SCR1* was used as an internal control. Error bars show the standard deviation (S.D.) calculated from two biological replicates, each with three technical replicates. (**C**) NuA3 HAT acetylates histones at the promoters of TREM genes. ChIP assay was performed as in Figure 3C. Error bars show the standard deviation (S.D.) calculated from three biological replicates, each with three technical replicates. *P < 0.05 (two-tailed unpaired Student’s *t* tests). (**D**) NuA3 binds to TREM genes. The average enrichment of Sas3 occupancy relative to the +1 nucleosome core particle (NCP) position analyzed using the data sets from Martin *et al.* (22). The plots represent the average enrichment of Sas3-HA for all genes (red) and for 544 TREM genes (purple). Blue indicates the average of log2 (IP/Input) values from mock sample.

Recently, we have shown that 544 genes exhibit stronger and faster repression during galactose incubation when the cells were previously exposed to the same carbon source. This phenomenon is termed TREM and is partly regulated by Rpd3L HDAC (12,46). The ING family protein, Pho23 in Rpd3L binds to H3K4me3 via its PHD finger and enhances histone deacetylation by Rpd3L to promote gene repression. Loss of this interaction by mutating tryptophan 305 to alanine (W305A) in the Pho23 PHD finger domain delays repression of 250 TREM genes during the second galactose incubation (12). However, the HAT with the opposite function remains to be identified. Since TREM genes have high levels of H3K4me3 recognized by the Yng1 PHD finger (12), NuA3 HAT may contribute to regulation of TREM. To test this hypothesis, gene expression patterns were analyzed in wild-types, *pho23* W305A, and *pho23* W305A/*yng1* W180A mutants. Cells were pre-cultured in media containing raffinose until mid-log phase and then shifted to galactose (120 min) for the first galactose exposure. After 120 min, the cells were transferred to glucose for 120 min and back to galactose (15 and 30 min) for the second galactose exposure (Figure 6A). As shown in Figure 6B, *RRN11* and *TEA1*, two TREM genes were downregulated rapidly upon the second galactose exposure in wild-type cells. However, this repression was delayed in the *pho23* W305A mutant that fails to bind to H3K4me3 (5,12). Interestingly, when the *yng1* W180A mutation was introduced in the *pho23* mutant, the kinetics of repression in the double mutant was similar to that in wild-type cells, indicating that the Yng1 PHD finger contributes to TREM (Figure 6B). Next, ChIP assay was performed to test whether the interaction between the Yng1 PHD finger and H3K4me3 is required for histone acetylation at promoters of TREM genes. H3K14 acetylation at *RRN11* and *TEA1* promoters was higher in the *pho23* W305A mutant than in wild-type cells (Figure 6C). Consistent with the gene expression patterns, H3 acetylation was reduced in double mutant than in *pho23* W305A mutant (Figure 6C). Sas3–6HA more strongly bound to TREM genes compared to all yeast genes (Figure 6D). These results suggest that NuA3 HAT and Rpd3L have antagonistic functions in regulation of histone acetylation and TREM.

## DISCUSSION

**Figure 7.**
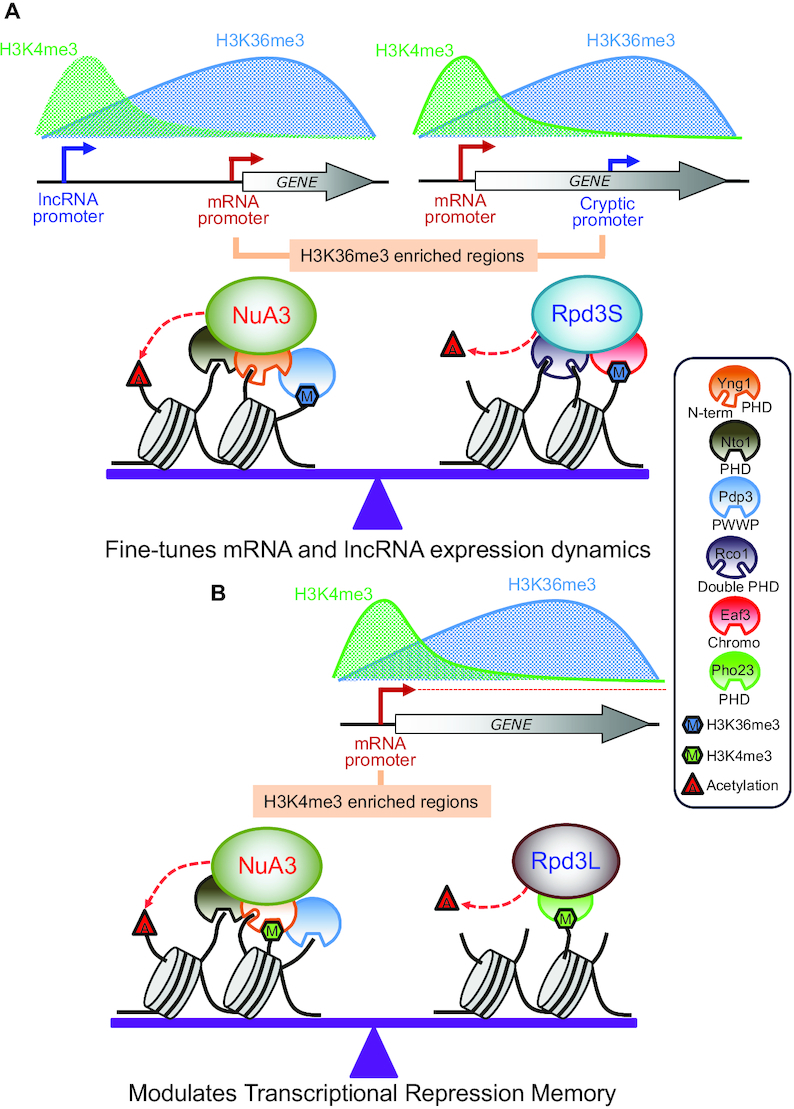
Models for regulation of gene expression by NuA3 HAT complex. (**A**) Opposing roles of NuA3 HAT and Rpd3S HDAC in regulation of histone acetylation at H3K36me3-enriched promoters. Transcription from an upstream lncRNA promoter targets H3K36me3 at the core promoter of mRNA target genes. In addition, transcription from the mRNA promoter places H3K36me3 in 3′ transcribed regions. Pdp3 of NuA3 HAT or Eaf3 chromodomain of Rpd3S HDAC modulates histone acetylation. The Yng1 N-terminal region, Nto1, and Rco1 may interact with unmodified histone tails to stabilize chromatin binding of NuA3 HAT or Rpd3S HDAC. Antagonistic function of NuA3 HAT and Rpd3S HDAC fine-tunes mRNA and lncRNA expression dynamics upon environmental changes. (**B**) NuA3 HAT may compete with Rpd3L HDAC at H3K4me3-enriched promoters to regulate histone acetylation and TREM. The Yng1 PHD finger of NuA3 HAT or Pho23 PHD finger of Rpd3L HDAC binds to H3K4me3 to control histone acetylation. Optimal level of histone acetylation at H3K4me3-enriched promoters is important for regulation of TREM.

Histone acetylation and deacetylation at promoters and within coding regions play important roles in initiation and elongation of RNA Pol II transcription (1,2). Unlike gene-specific activators or repressors that may function as on/off switches, chromatin regulators including HATs and HDACs tend to primarily affect the kinetics of gene induction or repression upon environmental changes (4,6,11,12,37,38). Optimizing gene expression dynamics is likely important for cells growing in the nature as they need to subsequently modify gene expression programs to adapt to rapidly changing environmental conditions. Although NuA3 HAT preferentially acetylates K14 and K23 of histone H3, its function in transcription remains largely unknown (16,29). Here, we show that NuA3 HAT contributes to the dynamics of mRNA and lncRNA expression promoting transcriptional activation or delaying gene repression upon carbon source shifts (Figure 7). Importantly, our data suggest that chromatin binding modules of NuA3 HAT may function together or independently to fine-tune gene expression dynamics by competing with two Rpd3 containing HDACs (Figure 7A and B).

Set2-mediated H3K36 and its downstream effector, Rpd3S HDAC, inhibit RNA Pol II elongation, initiation from internal cryptic promoter and histone exchange within transcribed regions (8,9,40,47). Furthermore, targeting of this pathway to mRNA promoters via overlapping lncRNA transcription downregulates mRNA transcription or delays gene induction (4,11,48). The data presented herein indicate that NuA3 HAT is required for full induction of mRNA and lncRNA transcription attenuated by the Set2-Rpd3S pathway (Figure 7A). This requires multiple chromatin binding domains of NuA3 HAT, the Yng1 N-terminal region (but not its PHD finger), Nto1 PHD finger and Pdp3 (Figures 3 and 4). Both the Yng1 N-terminal region and the Nto1 PHD finger likely mediate non-specific interactions between NuA3 HAT and chromatin as they recognize unmodified histones. Instead, the Pdp3 PWWP domain may bind to H3K36me3 at mRNA promoters targeted by lncRNA transcription or at cryptic promoters within coding regions (Figure 7A). These data suggest that promoters with high levels of H3K36me3 and low levels of H3K4me3 are fully induced by NuA3 HAT via the interaction between unmodified histones and NuA3 HAT mediated by the Yng1 N-terminal region and/or the Nto1 PHD finger, as well as by binding of the Pdp3 PWWP domain to H3K36me3. Pdp3 may also contribute to chromatin binding of NuA3 HAT via the association with unmodified histones. In addition, 82% of NuA3 target genes are overlapped by a lncRNA, including CUT, SUT or XUT classes, suggesting that NuA3 HAT might functionally interact with lncRNA transcription (49,50) (Figure 5E). It is important to note that only inducible promoters in mutants for the Set2-Rpd3S pathway are positively regulated by NuA3 HAT (Figure 4B and Supplementary Figure S3A). This could be due to the difference in target histones for these two complexes. NuA3 HAT selectively acetylates histone H3 K14 and K23, but Rpd3S deacetylates both histone H3 and H4 (14,47). The HATs required for transcription from constitutively active promoters remain to be identified.

We recently showed that many yeast genes display memory of their previous transcriptionally inactive state to allow effective suppression if they are no longer required for cellular functions. This TREM is important to rapidly turn off unnecessary genes and utilize cellular energy and the transcriptional machinery for genes supporting cell survival and fitness (12,46). Rpd3L HDAC binds preferentially to active promoters, likely via the interaction between H3K4me3 and the Pho23 PHD finger, to mediate histone deacetylation and TREM. Cells expressing a mutant version of Pho23, which fails to bind toH3K4me3, showed delayed repression of TREM genes upon environmental changes (12). Although interaction between the Yng1 PHD finger and H3K4me3 is important for targeting and for the function of NuA3 HAT (16,21), disruption of the interaction had no clear effect on mRNA and cryptic transcript levels repressed by the Set2-Rpd3S HDAC pathway (Supplementary Figures S2D and S3C). Strikingly, the H3K4me3–Yng1 PHD finger interaction was important for regulation of TREM genes (Figure 7B). Increased histone acetylation and delayed gene repression in the *pho23* mutant were reversed by mutating the Yng1 PHD finger (Figure 6B and C). These findings suggest that balanced acetylation and deacetylation at active promoters by NuA3 HAT and Rpd3L HDAC fine-tune the kinetics of gene repression upon environmental changes.

Chromatin modifying enzymes or complexes have multiple subunits that interact with distinct histone modifications. Although their exact functions remain elusive, NuA4 HAT includes Yng2 PHD finger and Eaf3 chromodomain recognizing H3K4me3 by Set1 and H3K36me3 by Set2, respectively (5,51). Rpd3S HDAC has an Eaf3 chromodomain and Rco1 PHD finger recognizing H3K36me3 by Set2 and unmodified histones, respectively (10,52,53). These multiple domains within a complex are believed to function together. For example, both Eaf3 and Rco1 are required for efficient chromatin binding and histone deacetylation by Rpd3S HDAC (10). Importantly, the present data suggest that the individual chromatin readers of NuA3 HAT seem to have distinct function in gene regulation. The Yng1 N-terminal region, the Nto1 PHD finger and Pdp3 of NuA3 HAT are necessary to counteract the Set2-Rpd3S HDAC pathway (Figure 7A). By contrast, the interaction between the Yng1 PHD finger and H3K4me3 promotes histone acetylation to delay gene repression mediated by Rpd3L HDAC (Figure 7B).

It has been suggested that NuA3 HAT exists in two distinct complexes: NuA3a containing Yng1 acting at promoters and NuA3b with Pdp3 functioning during transcription elongation (17). Consistent with this, peptide binding assays showed that although the common subunits, Sas3 and Nto1 were similar, the H3K4me3-bound form of NuA3 HAT had higher levels of Yng1 than H3K36me3-bound form (Figure 1B and C). The opposite pattern was observed for Pdp3. It should be noted that NuA3 HAT bound to peptides methylated on both K4 and K36 had higher levels of Yng1 or Pdp3 than the one bound to either H3K4me3 or H3K36me3 (Figure 1B and C). In addition, this binding was significantly reduced if *PDP3* or *YNG1* was deleted, indicating that a portion of NuA3 HAT includes both Yng1 and Pdp3 (Figure 1D and E). Unexpectedly, we found that the Yng1 N-terminal region was critical for the integrity of NuA3 HAT. In ΔN mutants, both Sas3 and Nto1 failed to bind to both H3K4me3 and H3K36me3. However, Pdp3 bound to H3K36me3 in both wild-type and ΔN mutants, indicating that loss of the Yng1 N-terminal region causes dissociation of Pdp3 from NuA3 HAT. In addition, co-immunoprecipitation assays showed that Yng1 N-terminal region was important for its association with NuA3 HAT (Figure 2). Based on these results, we propose that the Yng1 N-terminal region mediates the interaction between the core NuA3 HAT and two methyl-binders, Yng1 and Pdp3. An important question is what controls the formation of a distinct NuA3 HAT. Multiple subunits of NuA3 HAT are post-translationally modified, including Yng1 K42 ubiquitination, and phosphorylation at multiple sites of Sas3, Nto1 and Taf14 (54). Determining whether these modifications affect the integrity of NuA3 HAT will be important to understand the exact function of NuA3 subcomplexes in histone acetylation and transcription regulation.

## DATA AVAILABILITY

The RNA sequencing data sets that support the findings of this study have been deposited in the Gene Expression Omnibus with the accession code GSE148674.

## Supplementary Material

gkaa781_Supplemental_File
