# Supplementary material for: NuA3 HAT antagonizes the Rpd3S and Rpd3L HDACs to optimize mRNA and lncRNA expression dynamics
Source: Nucleic Acids Res. 2020 Oct 3;48(19):10753–67. doi: 10.1093/nar/gkaa781 (PMC7641726; doi:10.1093/nar/gkaa781)
Supplement: gkaa781_Supplemental_File [file gkaa781_supplemental_file.pdf]

Supplementary Figure 1

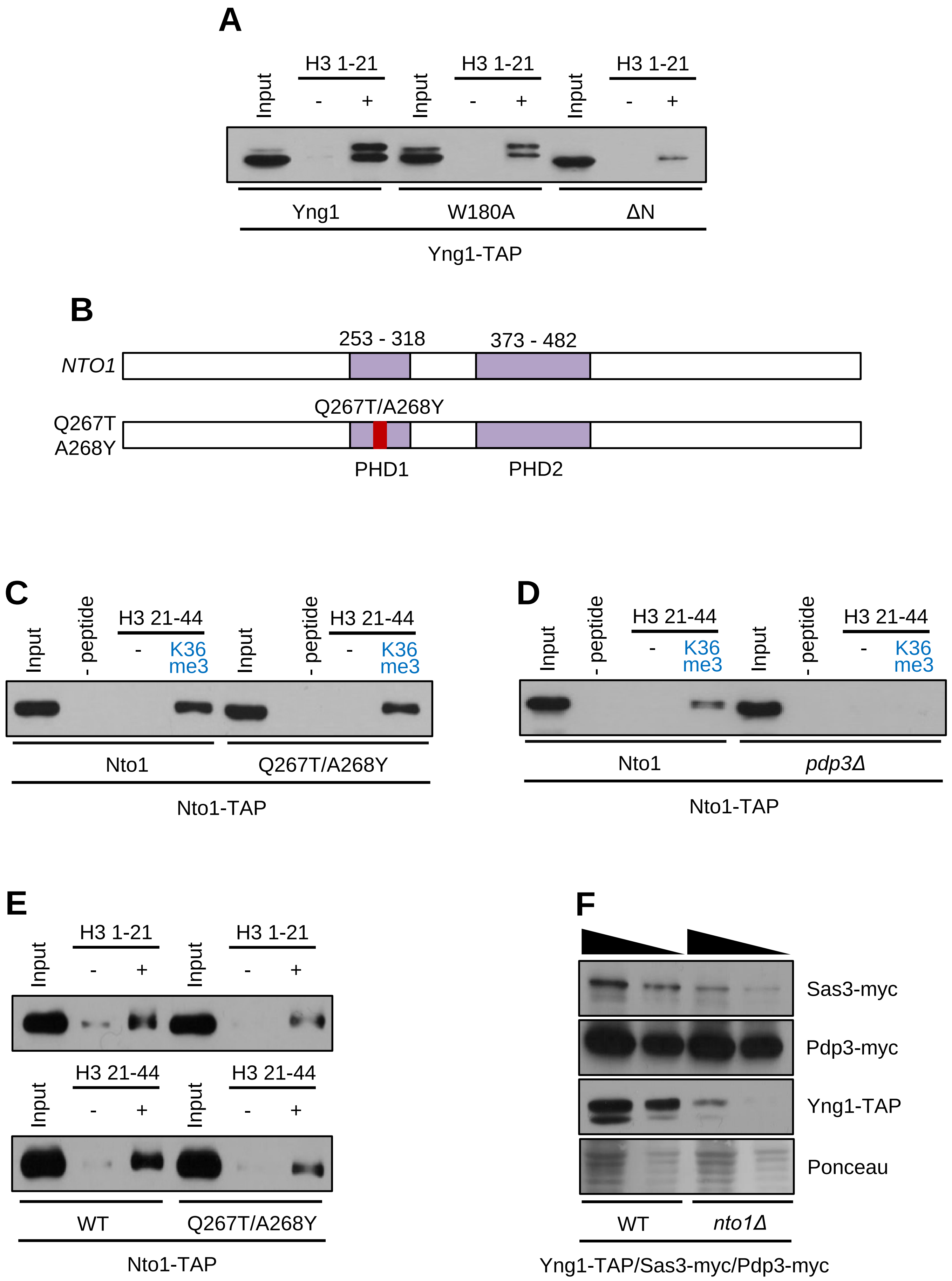

Supplementary Figure 2

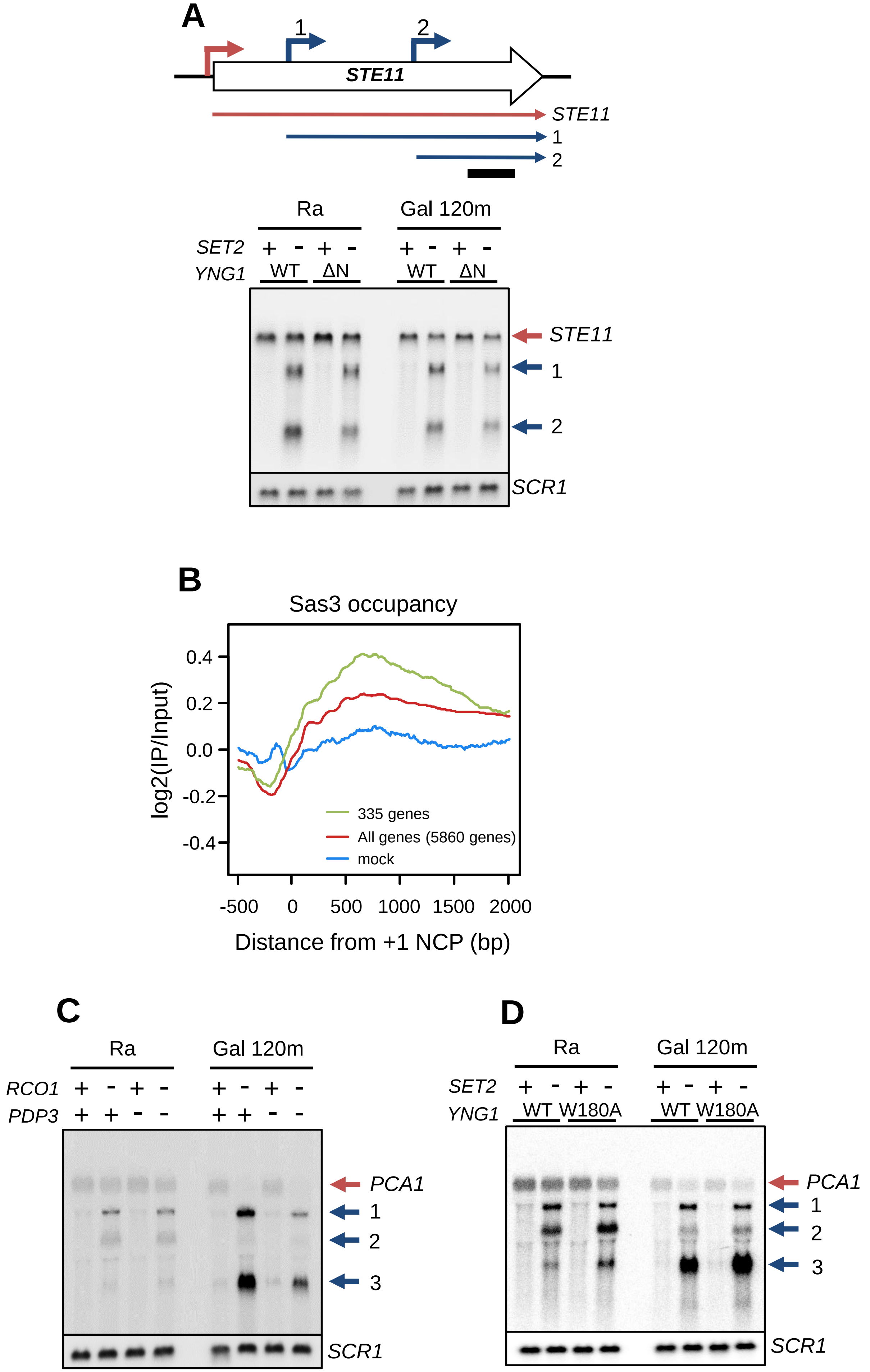

Supplementary Figure 3

A

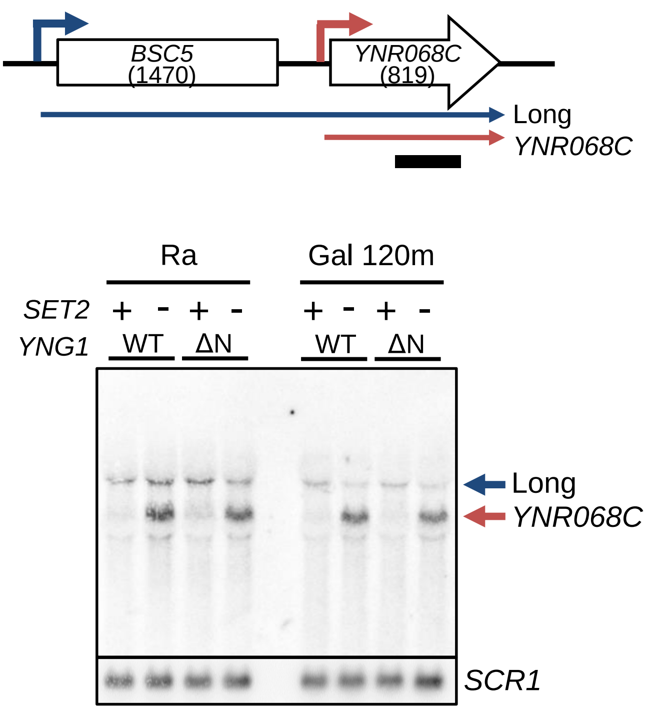

B

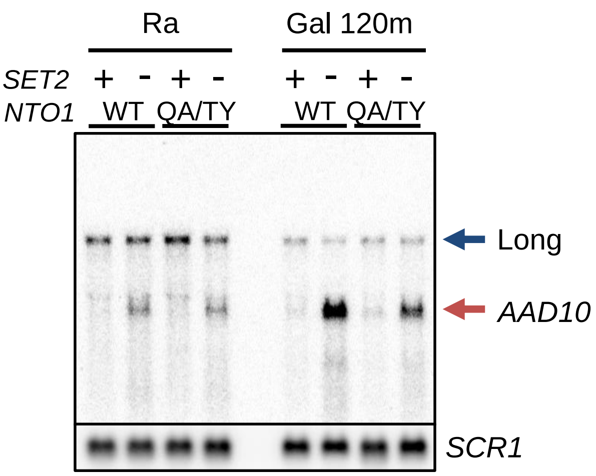

C

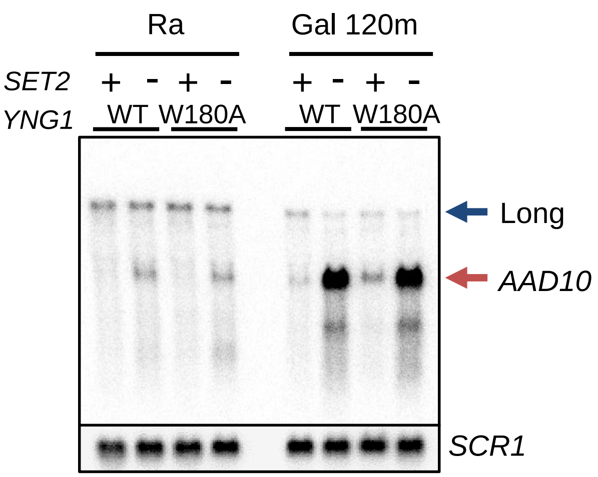

Supplementary Figure 4

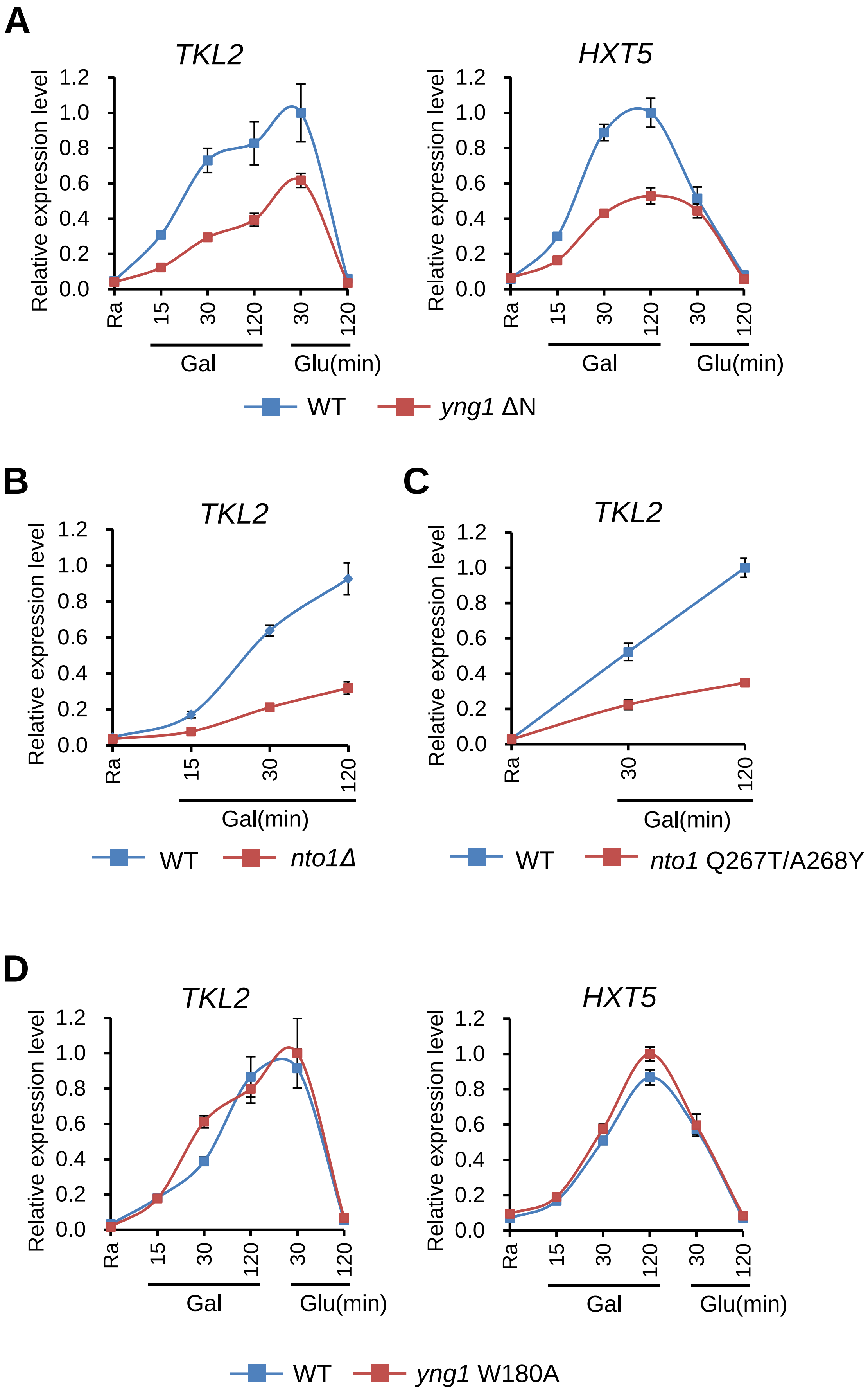

Supplementary Figure Legends

Supplementary Figure 1. Interaction between subunits of NuA3 HAT and histone peptides

(A) The Yng1 n-terminal region binds to unmodified histone tails. Histone peptide pulldown assays were performed using whole cell extracts from the indicated strains and 1 μg of histone peptides immobilized on magnetic beads in binding buffer containing 150 mM NaCl. Precipitated proteins were analyzed by immunoblot analyses with anti-TAP antibody. Two independent experiments showed the same results. **(B)** Schematic representation of the two PHD fingers of Nto1. The Q267T/A268A mutations in PHD finger 1 were created by the *delitto perfetto* strategy. **(C)** Mutation in the Nto1 PHD finger 1 does not affect its binding to H3K36me3. Histone peptide pulldown assay was carried out as in **A** using binding buffer containing 250 mM NaCl. **(D)** Interaction between Nto1 and H3K36me3 is abrogated in *pdp3Δ*. Histone peptide pulldown assay was done as in **C**. **(E)** Mutation in the Nto1 PHD finger 1 reduces its binding to unmodified histone tails. Histone peptide pulldown assay was performed using binding buffer containing 200 mM NaCl. **(F)** Loss of Nto1 reduces the levels of Sas3 and Yng1 proteins. Total extracts from the indicated strains grown in YPD were separated by SDS-PAGE and probed with the indicated antibodies.

Supplementary Figure 2. NuA3 HAT positively regulates inducible cryptic promoters

(A) Northern blot analysis of *STE11* cryptic transcripts with 3′-strand specific DNA probe (black box). The indicated cells were grown in synthetic complete (SC) medium containing raffinose (Ra) and shifted to SC-galactose media for 120 min (Gal 120m). Bottom panels show cryptic transcripts of *STE11* detected by northern blot analysis, which are schematicized at top. Red arrow is core promoter and blue arrows are cryptic promoters that produce short cryptic transcripts. *SCR1* was used as a loading control. Two independent experiments showed the same results. **(B)** The average enrichment of Sas3 occupancy relative to the +1 nucleosome core particle (NCP) position. Sas3 occupancy was analyzed using the data sets from Martin et al. (2017). The plots represent the average enrichment of Sas3-HA for all genes (red) and for 335 genes with inducible cryptic promoters (green). Blue indicates the average of log2 (IP/Input) values using mock sample. **(C)** Pdp3 is required for activation of galactose-inducible cryptic promoter of *PCA1*. Northern blot analysis of *PCA1* cryptic transcripts was performed as in **A**. **(D)** The Yng1 PHD finger domain is dispensable for regulation of galactose-inducible cryptic promoter of *PCA1*. Northern blot analysis of *PCA1* was performed as in **A**.

Supplementary Figure 3. NuA3 HAT and the Set2-Rpd3S pathway modulate mRNA induction via lncRNA transcription

(A) *YNR068C*, constitutively active in *SET2* deleting cells, is not regulated by NuA3 HAT. Northern blot analysis of *YNR068C* transcripts was performed with a 3′-strand specific DNA probe. The indicated cells were grown in synthetic complete (SC) medium containing raffinose (Ra) and shifted to SC galactose media for 120 min (Gal 120m). Bottom panel shows a lncRNA and *YNR068C* mRNA detected by northern blot analysis, which are schematicized at top. Red arrow indicates a core

promoter for *YNGR068C* mRNA, while blue arrow indicates a distal promoter that produces a lncRNA. *SCR1* was used as a loading control. **(B)** The Nto1 PHD finger is required for *AAD10* induction. Northern blot analysis of a lncRNA and *AAD10* mRNA was performed as in **A**. **(C)** The Yng1 PHD finger-H3K4me3 interaction is not required for *AAD10* induction. Northern blot analysis of a lncRNA and *AAD10* mRNA was carried out as in **A**.

Supplementary Figure 4. Fine-tuning of gene induction by NuA3 HAT

(A) NuA3 HAT is required for optimal induction of mRNA transcription. RNA samples from the time course experiments of wild type and *yng1* ΔN cells were analyzed by RT-PCR. *SCR1* was used as an internal control. Error bars show the standard deviation (S.D.) calculated from two biological replicates, each with three technical replicates. **(B-C)** Nto1 and its PHD finger positively regulate mRNA induction. Gene expression was analyzed as in **A**. **(D)** The Yng1 PHD finger-H3K4me3 interaction is not required for mRNA induction. mRNA expression was analyzed as in **A**.

Supplementary Table 1. Strains used in this study.

Strain	Genotype	Source or Reference
YSB787	MATa, bur1Δ::HIS3, ura3-52, leu2Δ1, trp1Δ63, his3Δ200, lys2Δ202 (pRS316-BUR1)	S.Buratowski
YSB2286	MATa, ura3Δ0, leu2Δ0, his3Δ1, met15Δ0, set2Δ::NatMX	S.Buratowski
YTK41	MATa, ura3Δ0, leu2Δ0, his3Δ1, met15Δ0, set2Δ::KanMX	Kim et al, 2016
YTK47	MATa, bur1Δ::HIS3, ura3-52, leu2Δ1, trp1Δ63, his3Δ200, lys2Δ202 (pRS316-BUR1), yng1Δ::KanMX	This study
YTK69	MATa, bur1Δ::HIS3, ura3-52, leu2Δ1, trp1Δ63, his3Δ200, lys2Δ202 (pRS316-BUR1), nto1Δ::KanMX	This study
YF336	MATa, ura3Δ0, leu2Δ0, his3Δ1, met15Δ0	Saccharomyces Genome Deletion Project
YTK84	MATa, ura3Δ0, leu2Δ0, his3Δ1, met15Δ0, PHO23-W305A	Lee et al, 2018
YTK95	MATa, ura3Δ0, leu2Δ0, his3Δ1, met15Δ0, YNG1-W180A	This study
YTK125	MATa, ura3Δ0, leu2Δ0, his3Δ1, met15Δ0, YNG1-ΔNterm	This study
YTK195	MATa, ura3Δ0, leu2Δ0, his3Δ1, met15Δ0, NTO1-Q264T/A265Y	This study
YTK198	MATa, ura3Δ0, leu2Δ0, his3Δ1, met15Δ0, YNG1-ΔNterm, set2Δ::KanMX	This study
YTK241	MATa, ura3Δ0, leu2Δ0, his3Δ1, met15Δ0, YNG1::TAP::HIS3	This study
YTK242	MATa, ura3Δ0, leu2Δ0, his3Δ1, met15Δ0, YNG1-W180A::TAP::HIS3	This study

YTK243	MATa, ura3Δ0, leu2Δ0, his3Δ1, met15Δ0, YNG1-ΔNterm::TAP::HIS3	This study
YTK244	MATa, ura3Δ0, leu2Δ0, his3Δ1, met15Δ0, NTO1::TAP::HIS3	This study
YTK245	MATa, ura3Δ0, leu2Δ0, his3Δ1, met15Δ0, NTO1-Q264T/A265Y::TAP::HIS3	This study
YTK278	MATa, ura3Δ0, leu2Δ0, his3Δ1, met15Δ0, YNG1-W180A, set2Δ::KanMX	This study
YTK280	MATa, ura3Δ0, leu2Δ0, his3Δ1, met15Δ0, NTO1-Q264T/A265Y, set2Δ::KanMX	This study
YTK304	MATa, ura3Δ0, leu2Δ0, his3Δ1, met15Δ0, trp1Δ::URA3	Lee et al, 2018
YTK385	MATa, ura3Δ0, leu2Δ0, his3Δ1, met15Δ0, pdp3Δ::KanMX	This study
YTK389	MATa, ura3Δ0, leu2Δ0, his3Δ1, met15Δ0, set2Δ::NatMX, pdp3Δ::KanMX	This study
YTK398	MATa, ura3Δ0, leu2Δ0, his3Δ1, met15Δ0, NTO1::TAP::HIS3, pdp3Δ::KanMX	This study
YTK469	MATa, ura3Δ0, leu2Δ0, his3Δ1, met15Δ0, PHO23-W305A, YNG1-W180A	This study
YTK550	MATa, ura3Δ0, leu2Δ0, his3Δ1, met15Δ0, trp1Δ::URA3, SAS3::18MYC::TRP1, YNG1::TAP::HIS3	This study
YTK551	MATa, ura3Δ0, leu2Δ0, his3Δ1, met15Δ0, trp1Δ::URA3, YNG1-ΔNterm::TAP::HIS3, SAS3::18MYC::TRP1	This study
YTK593	MATa, ura3Δ0, leu2Δ0, his3Δ1, met15Δ0, rco1Δ::LEU2	This study
YTK600	MATa, ura3Δ0, leu2Δ0, his3Δ1, met15Δ0, pdp3Δ::KanMX, rco1Δ::LEU2	This study
YTK697	MATa, ura3Δ0, leu2Δ0, his3Δ1, met15Δ0, trp1Δ::URA3, NTO1::18MYC::TRP1, PDP3::18MYC::LEU2, YNG1::TAP::HIS3	This study

YTK698	MATa, ura3Δ0, leu2Δ0, his3Δ1, met15Δ0, trp1Δ::URA3, NTO1::18MYC::TRP1, PDP3::18MYC::LEU2, YNG1-ΔNterm::TAP::HIS3	This study
YTK758	MATa, ura3Δ0, leu2Δ0, his3Δ1, met15Δ0, PDP3::TAP::URA3	This study
YTK776	MATa, ura3Δ0, leu2Δ0, his3Δ1, met15Δ0, trp1Δ::URA3, SAS3::18MYC::TRP1, YNG1::TAP::HIS3, pdp3Δ::KanMX	This study
YTK777	MATa, ura3Δ0, leu2Δ0, his3Δ1, met15Δ0, PDP3::TAP::URA3, yng1Δ::KanMX	This study
YTK844	MATa, ura3Δ0, leu2Δ0, his3Δ1, met15Δ0, trp1Δ::URA3, SAS3::18MYC::TRP1, YNG1::TAP::HIS3, PDP3::18MYC::LEU2	This study
YTK850	MATa, ura3Δ0, leu2Δ0, his3Δ1, met15Δ0, trp1Δ::URA3, SAS3::18MYC::TRP1, YNG1::TAP::HIS3, PDP3::18MYC::LEU2, nto1Δ::KanMX	This study

Supplementary Table 2. Oligonucleotides used in this study

Gene	**Sequences**
SCR1	up GAAGTGTCCCGGCTATAATA AA<br>low GACGCTGGATAAAACTCCCC
PCA1(Northern probe)	up TGGAGTTCATATCAATGAGGGAA<br>low CATAACGCAGAAGTATAGCTACA
PCA1(ChIP-cryptic promoter)	up TTCCTATCGTATTTGTTATTGCA<br>low TGTACTACAGTAAGTTTTCCTTC
AAD10(Northern probe)	up GACGGATATGGAGGTTAAAAT<br>low CACAATAAAAGATGCCTGCA
AAD10 (ChIP-promoter)	up TTTTGAGCTACTTGATGCTTTTT<br>low TACATCATACCCCTTATAATCC
RRN11(prom)	up GCATCGCTAACATCTAAAAAAGT<br>down AGGGACTTCAAACATGGTTTAT
RRN11(CDS)	up CATAACTGGTCGCTTGCATATAA<br>down TTGACACATCCATTGTAAGAAGT

TEA1(prom)	up CAAAAATGGAAAGTAGGTATGGG<br>down TTCATTCTTGTTTGGCCACA
TEA1(CDS)	up AGGACTAAACGATGATAATCCCA<br>down TTCTTGGAGTGATTGAAGAATCC
TKL2(CDS)	up GAAGACTGTTGTGGAACCCG<br>down TTCGGTAAATGCTTTTCCCAAC
HXT5(CDS)	up CAAACTCTGTCCAATGGAGG<br>down TAGCTCTTGCTAAGGACCGC
RAD28(Northern probe)	up ATAATGACGATCAAAGCGAT<br>down ATCATGGAACTAAACTGACA
RAD28(ChIP-cryptic promoter)	up CAGTTCTTTGCTTGTAGTGG<br>down TTCCCTGACGGGGTTAAATT
YNR068C(Northern probe)	up GAACACAAAAGATTGCAAAC<br>down TGAACTGACCTGATTCCATT
STE11(Northern probe)	up ATTCTGATGAGAATAATGAGC<br>down AGAAATTCTTTCCTTCTGACG
TEL VI	up TTTCGATCAAACTGCGTCAGG<br>down CGAGACAACGACAGGAAATAC
YNG1-W180A/pCORE	up TATCTTATGGGCCAATGGTGGCTTGTGATAATCCGGCTTGTCCCTTCGAG GAGCTCGTTTTCGACACTGG<br>down CATTTGCCCTTGGGAGCTTGTTTCAGGCCTACGCAACCATAATGGAACCA TCCTTACCATTAAGTTGATC
YNG1-W180A	up ATCCGGCTTGTCCCTTCGAGGCGTTCCATTATGGTTGCGTAGG<br>down CCTACGCAACCATAATGGAACGCCTCGAAGGGACAAGCCGGAT
YNG1-Nterm/pCORE	up GAACATCTCGCCAACGAAAACTCGGATTCGGATATCAGGT GAGCTCGTTTTCGACACTGG<br>down TAGTTCACATGGTAAATGATCCAAAGTGCTAAGGAAACTGT TCCTTACCATTAAGTTGATC
YNG1-Nterm deletion	TGCGCTAGCACATCCGCTTTACAAGGAAGTTTTTTTCGTCTACAGCAATGATAAGATCTCTTCGACTGATGCAA<br>ACAATCGACTTATTCAAGAATGAAGA
NTO1-Q264T_A265Y /pCORE	up TTATGAGCTATATGGTTCTGATGATGGAACAGGTTTATCGATGGACCAGG GAGCTCGTTTTCGACACTGG<br>down GACAATTGTATTCAGATTGTCACTGTCTGTGCCTAAACAAACTGCACACG TCCTTACCATTAAGTTGATC
NTO1-Q264T_A265Y	TTATGAGCTATATGGTTCTGATGATGGAACAGGTTTATCGATGGACACTTATTGTGCAGTTTGTTTAGGCACA<br>GACAGTGACAATCTGAATACAATTGTC

PHO23-W305A/pCORE	up TGGCATACGGGGAAATGGTGGGGTGTGATGGCGCAGACTGTGAGCTAGAA GAGCTCGTTTTCGACACTGG<br>down CACTTGCCCTTAGGTAGAGTTTCGAGTCCAATACATGGCAAATGGAACCA TCCTTACCATTAAGTTGATC
PHO23-W305A	TGGCATACGGGGAAATGGTGGGGTGTGATGGCGCAGACTGTGAGCTAGAAGCCTTCCATTTGCCATGTATT GGACTCGAAACTCTACCTA AGGGCAAGTG
